# Role of a Precision Biotic Fed to Dekalb White Laying Hens at Peak Production

**DOI:** 10.3390/ani15142095

**Published:** 2025-07-16

**Authors:** ElsiAnna Rodewald, Brooke Jasek, Li Zhang, Stacey Roberts, Cristiano Bortoluzzi, Pratima Adhikari

**Affiliations:** 1 Department of Poultry Science, Mississippi State University, Starkville, MS 39762, USA; 2 dsm-firmenich, Animal Nutrition and Health, North America, Belvidere, NJ 07823, USA; 3 Versova Management, Sioux Center, IA 51250, USA; 4 dsm-firmenich, Animal Nutrition and Health, 4303 Kaiseraugst, Switzerland

**Keywords:** egg mass, feed conversion, hen–day egg production, laying hen, precision biotic

## Abstract

The objective of this study was to determine the effects of two levels of a precision biotic (PB) within a reduced CP diet for performance, digestibility, and blood biomarkers in Dekalb White laying hens from 24 to 42 weeks of age. A total of four diets were fed: (1) an industry-standard positive control (PC) diet with a formulated CP level of 17.5%; (2) a negative control (NC) diet with a formulated CP level of 15.5%; (3) NC with 0.5 kg/ton PB (NC+PB1); (4) NC with 0.7 kg/ton PB (NC+PB2). The NC diet decreased hen–day egg production compared to the PC diet, and supplementation with both levels of PB led to intermediate levels of production. There was a similar trend for the feed conversion ratio (FCR), measured in kilograms of feed per dozen eggs. Feed cost was lower in the reduced CP diets compared to the PC. Apparent total tract digestibility was decreased with PB supplementation without differences in apparent ileal digestibility. The increased plasma uric acid levels in the NC+PB2 group suggest a shift in protein metabolism toward higher efficiency in ammonia detoxification. While more research is needed on PB, this research suggests that supplementation with PB in laying hens could potentially improve performance parameters.

## Simple Summary

In peaking age laying hens, performance, digestibility and egg quality parameters were studied. The objective was to compare the new feed additive, precision biotics in laying hens feed and observe how it can help in egg production and overall animal performance. A low protein diet and a standard protein diet with two levels of the feed additive were fed to the hens for 18 weeks. Research like this is important where the value of gut health and animal performance comes into the role of supporting overall animal health. While more research is needed on PB, this research suggests that supplementation with PB in laying hens could potentially improve performance parameters.

## 1. Introduction

The current study looked at the use of a precision biotic (PB) when implemented in a diet with industry-standard crude protein (CP) and a diet with reduced CP. The reduction in CP in layer diets can result in decreased nitrogen excretion [1,2]. High levels of nitrogen output are of environmental concern due to the potential for concentrated ammonia production [3]. Furthermore, reducing CP in layer diets can decrease feed costs; however, it may also result in significantly decreased profits [4,5]. Some studies have found that when the CP of diets is reduced, it can result in increased CP digestibility [2,6,7]. This is potentially an adaptive process to make up for the lower protein in the diet. However, if a reduced CP diet is not supplemented with synthetic amino acids (AA), then it can still result in lowered performance parameters such as average egg weight, hen–day egg production, and feed conversion ratio [4,8]. Therefore, supplementing feed additives that may further improve protein digestion could be economically and environmentally beneficial in a reduced CP diet.

Conventional prebiotic supplementation can lead to improved performance and a decreased incidence of disease in both layers and broilers; however, there may be some limitations to prebiotic efficacy in the poultry gut [9,10]. Specific prebiotics directly affect certain bacteria in the microbiome; for example, the addition of galactooligosaccharides in the diet primarily increases *Bifidobacteria* and *Lactobacilli* [11]. While these are considered beneficial bacteria, they can vary greatly among individual birds. A study conducted in humans and published by The Human Microbiome Project Consortium found that the taxonomic composition of the microbiome between healthy individuals can vary widely depending on location and living conditions [12]. Interestingly, despite the vast differences in bacterial taxonomy, the functional profile of the microbiome, as measured by its metagenome, was found to be much more stable.

Precision biotic (PB), also known as microbiome metabolic modulators [13], is a novel class of feed additive proposed and developed by dsm-firmenich within the last few years. Precision biotics are chemically synthesized glycans specifically designed and selected to modulate targeted microbiome functions. When prebiotics are consumed, they are utilized and broken down by the intestinal bacteria into sugar-based intermediates, which are used in a variety of microbiological functions [14]. Different from conventional prebiotics such as the previously mentioned galactooligosaccharides and others, PBs do not serve as a “food source” to the bacteria but instead can specifically signal core microbiome pathways of interest, such as short-chain fatty acid (SCFA) production, and nitrogen metabolism [13,15,16]. In this sense, PB could be fed to poultry flocks to target not only performance parameters but also other beneficial outcomes such as welfare and sustainability attributes [15,17,18]. Therefore, given the natural variations in bacteria that reside in the gut, in conjunction with the selective nature of prebiotics, it is reasonable to conclude that the use of PBs would deliver higher consistency in terms of beneficial outcomes.

By harnessing the full potential of the microbiome functions, PBs can act as modulators of specific pathways that may include the modulation of assimilation pathways of amino acids (AAs), putrefactive pathways, and ammonia production [15,19]. Current PBs are composed of a selected group of synthesized glycans that act as metabolic modulators. When an animal consumes carbohydrates, oligosaccharides are broken down into glycans, which are sugar-based intermediates used in a variety of biological functions, including glycoprotein synthesis [14]. By providing the intermediates necessary for further bacterial involvement in these pathways, PB may more efficiently modulate bacterial genes responsible for specific metabolic functions [20]. Previous research conducted with human pathogens has demonstrated that synthetic glycans can inhibit pathogenic bacteria, and a study with mice found that they can alter the taxonomic composition of the microbiome [21,22]. Through the modification of the metabolic pathways and microbiome of the bird, PBs have the potential to lead to increased production and efficiency in the bird, better protein utilization, as well as a decrease in ammonia concentrations due to greater nitrogen incorporation [23].

Several studies conducted in broilers on the components of the PB studied herein, or the actual PB itself, showed that its supplementation increased SCFA production and decreased litter pH and ammonia production [15,16]. Supplementation with PBs in broilers decreased ammonia concentrations in litter but did not affect total nitrogen content, suggesting that nitrogen metabolism was indeed shifted [15]. This was further supported by an increase in arginine-N-succinyl transferase in supplemented birds, suggesting improved AA catabolism [15]. Additionally, Blokker et al. [24] gave evidence supporting the hypothesis that PBs affect protein metabolism, though they reported effects on gene expression rather than microbiome composition. Enterically challenged birds were given treatments with or without PBs and compared to unchallenged birds. The unchallenged birds had the highest levels of gene expression for the peptide transporter PepT1, to which the birds supplemented with PB were statistically similar [24]. The increased mRNA abundance of PepT1 has been associated with increased levels of digestible AA in broiler chicks. This relationship between PepT1 and CP, applied to the reported upregulation of PepT1 in response to PB by Blokker et al. (2022) [24], suggests that PBs may have an impact on protein absorption in the small intestine [25].

On the production side, birds fed with PBs showed improved body weight gain, feed intake, and feed conversion ratio [13,15,16,19]. Furthermore, Bortoluzzi et al. (2023) and Yan et al. (2023) found that supplementation with PBs at concentrations of 1.1 and 0.9 kg/ton resulted in a higher efficiency index, which demonstrated the economic potential of PBs in a standard CP broiler diet [16,19].

Prior to this research being conducted, there had been no published studies conducted with PB in laying hens, but since then, a study in which 0.9 kg/ton of PBs was supplemented to free-range laying hens from 17 to 72 weeks of age has been published. The authors found that supplementation with PBs resulted in lower mortality rates, better feed conversion, and improved gut integrity in early and mid-lay phases [18].

While PBs have been evaluated in standard broiler diets, there have been no published studies evaluating their effects in reduced CP diets in laying hens on digestibility and blood biomarker parameters. Therefore, the objectives of this study were to determine the effect of two levels of PBs in conjunction with a reduced CP diet for performance parameters, CP digestibility, and blood biomarkers in laying hens from 24 to 42 weeks of age using Dekalb White layers in the layer phase one. This study hypothesized that supplementation with precision biotics in a reduced crude protein diet would improve egg production and influence feed efficiency in Dekalb White laying hens.

## 2. Materials and Methods

All animal care procedures were approved by the Mississippi State University Institution of Animal Care and Use Committee (IACUC #22-366).

### 2.1. Pre-Experimental Period

A total of 448 22-week-old Dekalb pullets were obtained from chicks hatched at a commercial hatchery and raised at a commercial table egg facility. Hens were transported from the facility to the Mississippi State Poultry Research Unit at 17 weeks of age. After adapting to feed and environment for five weeks, hens were further adapted for a two-week pre-experimental period before the start of feeding experimental diets. Each cage (758 cm^2^) was stocked with four pullets as per the Mississippi State University animal welfare guideline policy. During this period, feed and water were provided ad libitum. A standard layer phase one mash diet formulated according to the Dekalb White Management Guideline was fed. Egg production was recorded once daily and, at the start of the second week, average egg weight (AEW) was recorded to obtain a baseline comparison between the different treatment groups.

### 2.2. Experiment Design, Birds, and Feed

The experiment was conducted in a randomized block design where the location of the cage, both within the house and on the levels of the A-frame, was considered the blocking factor. Each of the cages was considered an experimental unit with four birds per cage. In total, there were 112 hens per treatment allocated into fourteen blocks, each containing two replicates of each of the four treatments. Thus, there was a total of 28 replicating cages per treatment. Within the blocks, one each of the treatment replications was on a top-level cage and the other was on a bottom level.

Four dietary treatments were evaluated in this experiment. The results reported in this paper are part of a larger experiment containing nine dietary treatments. Feed was formulated as a layer phase one mash diet throughout the experimental period. Diets, calculated values, and analyzed values are outlined in Table 1. Feed was formulated based on NIR analysis of ingredients, and analyzed values for feed were reported using proximate analysis. Specifications met or exceeded the Dekalb White Management Guideline for all nutrients except CP and digestible amino acids (dAAs). Most of the limiting AAs (Trp, Thr, Arg, Ile, TSAA, Met, Val) were formulated on the ratio of dLys. Treatment 1 was designated as the positive control diet (PC), which was an industry-standard diet formulated to 17.5% CP and 0.90% dLys that met all AA recommendations. The other three treatments were based on a reduced CP diet that was formulated to 15.5% CP and 0.85% dLys without or with PBs (Symphiome^®^, dsm-firmenich, Kaiseraugst, Switzerland). In the reduced CP diets, all AAs were balanced and expressed as the ratio of dLys. The reduced CP diet without any PB supplementation was considered the negative control (NC; treatment 2). The other two reduced CP diets were supplemented with either 0.5 (treatment 3) or 0.7 kg/ton (treatment 4) of PBs and were considered NC+PB1 and NC+PB2, respectively. Treatment diets were fed between 24 to 42 weeks of age following the adaptation period from 22 to 24 weeks of age.

The birds were weighed at the beginning of the trial period and the end of the experimental period. Change in body weight (CBW) was calculated by finding the difference between the ending body weight and the beginning body weight in grams. Birds were given ad libitum access to water. Every pen had a corresponding feed bucket of known weight that was filled with enough feed to last a week at a calculated feed intake of ~115 g per bird per day. Feed was added from the bucket to customized feeders four times a week (Monday, Wednesday, Friday, and Saturday). The remaining feed at the end of the week was weighed back every Wednesday at 7:00 a.m., and all feed weigh back was completed within a two-hour window. Feed intake was calculated weekly by taking the difference between the beginning and ending bucket weight in grams. Feed intake was reported in terms of both grams per bird per day (g/b/d) and pounds per hundred birds per day (lbs/100). The equation for g/b/d is shown below:

(1) $$g/b/d=\frac{bucket\;weight\;at\;beginning\;of\;week\left(g\right)-weight\;at\;end\;of\;week\left(g\right)}{\#\;of\;hens\;*\;7\;days}$$

The equation for lbs/100 is shown below:

(2) lbs/100=((g/b/d)/453.592)∗100

### 2.3. Egg Production and Performance Data

Egg data were collected daily at 3:30 p.m. within an hour window. The number of eggs and external egg flaws were recorded daily for each cage. To calculate production, weekly hen–day egg production (HDEP) was used. The number of eggs produced in each cage in a week was divided by the total number of hen–days within that cage during that week. The equation for weekly HDEP is shown below:

HDEP = total # of eggs for the week/(# of hens ∗ 7 days)

Egg flaws were recorded as unsalable eggs (UEs) and included any egg that was shell-less or soft-shelled, extremely small, extremely large, dirty, cracked, bloody, or had very visible ridges or calcium deposits. Average egg weight (AEW) was calculated weekly for each experimental unit. Every Wednesday, eggs from each cage were weighed together on a tared egg flat to find the total egg weight for that cage. The total weight was divided by the number of eggs collected to find the AEW. Feed conversion ratio was calculated in terms of both grams of feed per gram of egg (gf/ge) and kilograms of feed per dozen eggs (kg/doz). The equation for gf/ge is shown below:

(3) gf/ge=grams of feed consumed/(AEW ∗ total # of eggs for the week)

The equation for kg/doz is shown below:

(4) $$kg/doz=\frac{grams\;of\;feed\;consumed}{1000}/\frac{total\;\#\;of\;eggs\;for\;the\;week}{12}$$

### 2.4. Apparent Total Tract Digestibility and Apparent Ileal Digestibility of CP

One week prior to the end of the study, one bird from one replication for seven blocks was randomly selected and placed into an individual cage for excreta collection within the same house. One replication from each treatment was chosen from seven blocks at random to receive their feed with a 0.5% titanium dioxide (TiO_2_) marker. Feeding and egg-collecting schedules remained the same. Fecal collection trays were placed underneath the cages of the birds. The feed with the marker was supplied for a total of 10 h, after which, the excreta was collected from the trays. The excreta samples were later weighed, oven-dried for 24 h at 105 °C, and then re-weighed to find the moisture content. The dried excreta samples along with the feed samples containing TiO_2_ were shipped to an outside lab (ATC Scientific, North Little Rock, AR) for analysis of crude protein, as well as TiO_2_. The apparent total tract digestibility (ATTD) of crude protein was calculated by the following equation [26].

(5) $$\%ATTD\;CP=\frac{({\frac{CP}{{TiO}_{2}})}_{Diet}-({\frac{CP}{{TiO}_{2}})}_{Excreta}}{({\frac{CP}{{TiO}_{2}})}_{Diet}}*100$$

Digesta samples were collected from the birds that received the TiO_2_ feed. Hens were euthanized by CO_2_ inhalation and ileum samples were collected for CP and AA analysis.

Ileal contents were collected beginning at the distal end of the ileum from approximately three cm below the Meckel’s diverticulum to approximately three cm proximal to the ileocecal junction. The digesta was gently flushed from the ileum with distilled water, frozen, then freeze-dried. The freeze-dried samples along with the feed samples containing TiO_2_ were sieved and shipped to an outside lab (ATC Scientific, North Little Rock, AR, USA) for analysis of CP and TiO_2_. Crude protein was analyzed according to the methodology described in AOAC 968.06 [27]. Titanium dioxide was analyzed according to a modification of the method described in AOAC 2006.03 [28]. The apparent ileal digestibility (AID) of CP was calculated by the following equation [26].

(6) $$\%AID\;CP=\frac{({\frac{CP}{{TiO}_{2}})}_{Diet}-({\frac{CP}{{TiO}_{2}})}_{Digesta}}{({\frac{CP}{{TiO}_{2}})}_{Diet}}*100$$

### 2.5. Blood Biomarkers

At 42 weeks of age, lithium–heparin syringes were used to draw 1 mL of blood from the wing vein of each of the birds that had been given titanium dioxide feed (seven birds per treatment). The blood was then injected into an avian/reptilian Vetscan cartridge and was analyzed by a Vetscan VS2 machine (Abaxis, Inc., Union City, CA, USA) with the avian/reptilian profile according to the manufacturer’s instructions. Levels of plasma sodium (Na), potassium (K), phosphorus (P), aspartate aminotransferase (AST), uric acid (UA), globulins (Glob), albumins (ALB), total protein (TP), glucose (Glu), and creatine kinase (CK) were determined.

### 2.6. Economic Analysis

Economic analysis was conducted from 24 to 41 weeks of age. The cost of feed was held constant throughout the study period and the values per ton are as follows: PC = 556.75 USD; NC = 531.51 USD; NC+PB1 = 533.24 USD; NC+PB2 = 533.93 USD. Prices for table eggs which included weight grades of extra-large, large, and medium were obtained weekly starting from 14 October to 10 February from the United States Department of Agriculture Agricultural Marketing Service shell eggs report for the southeast region [29]. Eggs that fell outside this range were considered discarded. The cost per hundred hens per day was determined by multiplying the cost of a pound of feed for the respective treatment by lbs/100. Then, income per hundred hens per day was found by first finding the weight grades of the eggs for each pen using the AEW, then multiplying the HDEP by the price for the respective grade. The cost was subtracted from the income. Values were reported in dollars per hundred hens per day (USD/100/d).

### 2.7. Statistical Analysis

Outliers for performance data were removed using Cook’s D-value. The production data and economic analysis data were analyzed in a mixed model using the PROC GLIMMIX procedure of SAS 9.4 (SAS Institute Inc., Cary, NC, USA). The data were analyzed as repeated measures, where the type of variance–covariance structure was defined as unstructured. Degrees of freedom were determined using the Kenward Roger estimation and least significant means for each treatment were analyzed for each treatment overall and within each week with adjustments by the Tukey–Kramer procedure. The following population model was used to analyze the production data:

(7) $$\gamma_{ijk}=\mu +\tau_{i}+{\delta \tau }_{\left(i\right)j}+t_{k}+{\left(\tau \;*\;t\right)}_{ik}+{\varepsilon \prime }_{ijk}$$

where:

γ_ijk_ = production data;
μ = mean of the whole population;
τ_i_ = effect of treatment;
t_k_ = effect of weekly measurement from 24 to 41 weeks;
(τ ∗ t)_ik_ = effect of interaction between treatment and week;
δτ_(i)j_ = random error of repeated measurements with mean 0 and covariance σ_ij_;
ε’_ijk_ = random error with mean 0 and variance σ_i_^2^.

The model was constructed under the assumption that these data are normally distributed. The total variation of the model was composed of variation due to repeated measurements and variation due to random error. The adaptation, digestibility, and blood biomarker data were analyzed in a one-way ANOVA based on a two-sided alternative hypothesis using the PROC GLM procedure of SAS 9.4 (SAS Institute Inc., Cary, NC, USA). Means with significant (*p* ≤ 0.05) treatment effects in ANOVA were subject to post hoc means separation using Fisher’s least significant difference. Pairwise comparisons using Student’s *t*-test are displayed for digestibility and blood biomarker data.

## 3. Results

### 3.1. Egg Production and Performance Data

For the adaptation period, there were no differences between treatments. However, to ensure that there was no covariance, a mixed model with repeated measures was utilized to analyze the production data. Egg production data analyzed from the entire study are presented in Table 2. The lowest HDEP was observed among hens fed the NC diet of 15.5% CP without PB.

Both inclusion levels of PBs had production that was not significantly different compared to the NC diet (98.04% and 98.20% compared to 97.58%), nor to the PC diet (98.75%). However, the HDEP for the PC diet was significantly higher than that of the NC diet (*p* = 0.0028). There was a trend in AEW in which the PC diet had the highest AEW (59.17 g) and the NC had the lowest (58.26 g) compared to the other treatments (*p* = 0.0935). There were no significant differences for UE (*p* = 0.8631).

### 3.2. Feed Intake, Feed Conversion Ratio, and Body Weight

Feed intake (g/b/d and lbs/100) and FCR (gf/ge and kg/doz) data for the entire study are presented in Table 3. There were no significant differences between treatments for gf/ge (*p* = 0.2355). There was a trend towards significance in kg/doz in which the PC had the lowest FCR and the NC had the highest compared to the other treatments (*p* = 0.0624). The numerical differences grew more pronounced in the later weeks compared to the initial weeks, suggesting the need for a study in later phases of laying. There were no significant differences between treatments for g/b/d or lbs/100 throughout the study (*p* = 0.7042; *p* = 0.7043).

### 3.3. Apparent Total Tract Digestibility and Apparent Ileal Digestibility of CP

The results for digestibility are presented in Table 4. No significant differences were seen between treatments for the AID of CP (*p* = 0.2651). However, the NC diet (55.95%) had the highest ATTD of CP followed by the PC (43.15%; *p* = 0.0462). Supplementation with both levels of PBs (41.32%; 37.69%) decreased the ATTD of CP from the high levels shown with the NC and brought it to similar levels as the PC, suggesting the potential for PBs to help adapt digestibility in a reduced CP diet by modulating microbial pathways in the ceca related to nitrogen utilization [30].

We hypothesized that HDEP and AEW would increase with supplementation with PBs in a reduced CP diet due to increased CP digestibility. However, while HDEP was numerically increased, there were no differences in the AID of CP, which is supported by research in broilers supplemented with 0.9 kg/ton [30]. Additionally, the ATTD of CP was increased in the NC diet compared to those supplemented with PB. The digestibility of CP in a decreased CP diet has shown mixed results in the literature [2,7,31]. Most studies in poultry have looked exclusively at AID due to the demonstrated variability that arises in ATTD values from bacterial fermentation in the ceca [26]. However, Ding et al. (2016) reported that in broilers, the ATTD of CP was decreased with decreased CP levels [31]. Conversely, increased ATTD of CP in a decreased CP diet was reported by Heo et al. (2023) [2]. Similarly, Awad et al. (2015) reported that the AID was significantly increased when the CP of a diet was reduced from 19.5% to 13.5% but was fortified with crystalline AA [7]. The authors suggested that this could have been due to either improved AA digestibility or because of improved efficiency. In the standard CP diet, AAs in excess of the birds’ requirements may have ended up being excreted.

It was expected that PB would increase digestibility of CP due to an increase in nitrogen assimilation into products such as SCFA, BCFA, and others. Two previous studies have calculated the microbiome protein metabolism index (MPMI) of the microbiome, which gives a ratio of the metabolic processes of beneficial microbial protein assimilation to the metabolic processes of undesired protein putrefaction. In both these studies, the MPMI index was increased by PB supplementation, suggesting improved protein assimilation [16,19]. In this research, the significant decreases in ATTD from the NC when a PB was added along with the lack of differences in AID suggests that the supplementation with the PB increased the endogenous production of protein, specifically after the ileocecal junction, or microbial protein production in the caeca.

The PC group had an improved HDEP compared to the NC, which may be explained by the fact that excess CP that is not absorbed as AAs is broken down into BCFAs by proteolytic enzymes [32]. BCFAs are first alpha-oxidized, then beta-oxidized to form acetic acid, an important component for the Krebs cycle to produce ATP [33]. While the NC diet may have met AA requirements, the lower energy production from protein may have resulted in a lower HDEP. When the PB was supplemented, potential increases in SCFAs such as acetic acid may have led to an increase in the production of ATP and other metabolites from the Krebs cycle, which may have influenced the production capabilities of the hens [32,33].

### 3.4. Blood Biomarkers

The results for blood biomarkers are found in Table 5. Treatment with NC+PB2 resulted in higher UA (2.900 mg/dL) than the PC, NC, and NC+PB1 (1.972; 1.644; 1.779; *p* = 0.0312). Similarly, NC+PB2 had higher P (4.885 mg/dL) compared to the PC, NC, and NC+PB1 (3.971; 3.851; 3.420; *p* = 0.0375).

The elevated uric acid levels in a lower CP diet with lower digestibility, along with a similar feed intake among all groups, suggest that the conversion of ammonia to uric acid was increased in the NC+PB2 birds [34]. Uric acid is an antioxidant and is part of the nitrogen excretion system employed by birds [33,35]. Petranyi et al. (2024) reported that laying hen mortality due to smothering was significantly decreased in a test group supplemented with 0.9 kg/ton PBs compared to a control group, which may suggest that these birds were better able to tolerate smothering-associated stressors [18]. It should be noted, however, that a recent study conducted by Bortoluzzi et al. (2024) [30] with broilers found that those given PBs at 0.9 kg/ton from the starter through the finisher phase had lower plasma UA concentrations than those fed a control diet. However, the average level of UA in the broilers supplemented with PBs was nearly identical (2.91 mg/dL) to the UA level of the hens supplemented in our study (2.90 mg/dL), and PB supplementation was tested in starter, grower, and finisher diets with recommended CP levels (21%; 19%; 18%) [28]. Additionally, Bortoluzzi et al. (2024) found that supplementation with PBs in broilers fed a corn-based diet altered the abundance of genes related to nitrogen metabolism [30]. A possible explanation for the differences in UA results between this study and Bortoluzzi et al. (2024) is that levels of 0.7 to 0.9 kg/ton of PBs helped to modulate nitrogen metabolism to result in similar levels of UA regardless of dietary protein content [30]. Further research in this area may include testing how supplementation with the PB affects performance when the birds are exposed to environmental stressors such as heat or cold compared to a control and testing the difference between PB supplementation in both full and reduced CP diets. The other blood biomarker that was significantly different between treatments was plasma P. Like plasma UA, it increased in NC+PB2 without also showing increased feed intake, suggesting improved phosphorus metabolism. Certain bacteria have roles in phosphorus release, so it is possible that the PB was able to modulate pathways related to these bacteria [36]. However, more research is needed in this area.

**Table 5:** Effect of varying levels of a precision biotic in a reduced crude protein diet on concentration of plasma blood biomarkers.

Trt ^1^		TP ^2^ (g/dL)	AST ^3^ (U/L)	CK ^4^ (U/L)	UA ^5^ (mg/dL)	Glu ^6^ (mg/dL)	P ^7^ (mg/dL)	Alb ^8^ (g/dL)	Glob ^9^ (g/dL)	K ^10^ (mmol/L)	Na ^11^ (mmol/L)
Reference		3.9–7.0 ^13^	118–298 ^13^	107–1780 ^13^	0.9–8.9 ^13^	208–279	1.6–7.2 ^13^	1.5–3.3 ^13^	1.6–4.3 ^13^	3.9–6.5	141–159
PC		5.414	185.9	1215	1.972 ^b^	235.2	3.971 ^b^	2.617	2.737	5.258	148.7
NC		5.355	161.3	804	1.644 ^b^	250.4	3.851 ^b^	2.795	2.547	5.003	147.7
NC+PB1		5.319	179.0	1338	1.779 ^b^	236.9	3.420 ^b^	2.543	2.782	5.038	147.0
NC+PB2		5.443	197.5	1816	2.900 ^a^	244.4	4.885 ^a^	2.631	2.823	5.243	148.3
SEM ^12^		0.175	13.24	287.1	0.311	6.300	0.338	0.082	0.129	0.147	1.505
*p*-value		0.9550	0.2901	0.1262	0.0312	0.3699	0.0375	0.3416	0.5240	0.4605	0.8663
Pairwise Comparisons (*p*-Values)											
PC vs.	NC	0.8127	0.2054	0.3282	0.4659	0.1058	0.8044	0.1436	0.3128	0.2345	0.6276
	NC+PB1	0.7173	0.7290	0.7719	0.6806	0.8553	0.2855	0.5495	0.8128	0.3257	0.4588
	NC+PB2	0.8986	0.4980	0.1119	0.0318	0.2669	0.0485	0.8917	0.5985	0.9394	0.8240
NC vs.	NC+PB1	0.9074	0.4508	0.3045	0.8059	0.2343	0.4722	0.0964	0.3123	0.8897	0.8099
	NC+PB2	0.7258	0.0706	0.0256	0.0117	0.5026	0.0458	0.1763	0.1512	0.2606	0.7740
NC+PB1 vs.	NC+PB2	0.6382	0.3585	0.2681	0.0273	0.4356	0.0100	0.4761	0.8299	0.3572	0.5793

^a,b^ Means within a column lacking a common superscript differ (*p* ≤ 0.05) as per least significant difference. Bolded *p*-values are significant. Abbreviations: ^1^ Trt = treatment; PC = 17.5% CP; NC = 15.5% CP; NC+PB1 = 15.5% CP + 0.5 kg/ton precision biotic (PB); NC+PB2 = 15.5% CP + 0.7 kg/ton PB; ^2^ TP = total protein; ^3^ AST = aspartate aminotransferase; ^4^ CK = creatine kinase; ^5^ UA = uric acid; ^6^ Glu = glucose; ^7^ P = phosphorus; ^8^ Alb = albumen; ^9^ Glob = globulin; ^10^ K = potassium; ^11^ Na = sodium; ^12^ SEM = standard error of means; ^13^ Board et al., 2018 [37]; n = 7 observations per treatment.

There are a couple of different interpretations that can be considered as to why the CK levels of NC+PB2 may be numerically elevated in comparison to the NC. The only biomarker that is outside the reference range established by Board et al. (2018) is CK [37]. Elevated CK levels, especially in conjunction with elevated AST levels, may be indicative of muscle metabolism [38]. In the absence of sufficient dietary protein, hens may break down muscle tissue for egg production [39,40]. While the NC group slowed egg production to match the reduced CP in their diet, the HDEP of NC+PB1 and NC+PB2 was not significantly different than the PC. Furthermore, the ATTD of CP results suggest that the addition of PBs to a reduced CP diet may decrease digestibility in the lower intestinal tract in comparison to a reduced CP diet alone. However, even though the birds fed 0.7 kg/ton of PBs may have had lower CP digestibility while maintaining a high level of production, it should not be assumed that the birds depleted muscle to maintain production, as this is not necessarily reflected in the body weight data. There were no significant differences in body weight between the NC and NC+PB2, as shown in Table 3. In fact, numerically, NC+PB2 had a greater increase in body weight compared to the NC. Additionally, the levels of AST, a marker of muscle deterioration, for both the NC and NC+PB2 are well within the reference range established by Board et al. (2018) [37]. Given the limited sample size for the blood biomarker data, it is possible that these differences within the normal range are due to normal variation among birds; therefore, it is difficult to make conclusive statements regarding how the biomarkers were elevated. Further research in this area could include a comparison of the ratio of breast muscle weight to body weight across treatments and/or weekly measurements of changes in body weight over a longer trial period to better determine if PB may impact muscle metabolism.

### 3.5. Economic Analysis

The results of the economic analysis are shown in Table 6. Both NC+PB1 and NC+PB2 had significantly decreased feed costs from the PC by −0.28 USD/100/d and −0.24 USD/100/d, respectively (*p* < 0.0001). There were no significant differences for income or for feed cost subtracted from income. Based on the numerically decreased kg/doz and the economic analysis data, supplementation with PB may have the ability to help producers save on costs; however, more research needs to be done.

## 4. Conclusions

The reduced CP diet had a lower HDEP than the standard CP diet, but supplementation with PBs in the reduced CP diet resulted in an HDEP that was not different than that of the standard CP diet. The PB numerically decreased kg/doz in a reduced CP diet. There were no significant differences between NC+PB1 and NC+PB2 for any of the parameters measured except for the blood biomarkers. Supplementation with PBs decreased the ATTD of CP, which goes against our hypothesis; however, it also promoted higher concentrations of plasma uric acid and plasma phosphorus, which may indicate more efficient metabolic pathways for ammonia excretion. While more research is needed on PBs, this research suggests that supplementation with PBs in laying hens has the potential to be economically advantageous.

## Figures and Tables

**Table 1:** Ingredient percentages and calculated values for the treatment diets.

	Treatments ^1^			
Ingredients (%)	PC	NC	NC+PB1	NC+PB2
Corn	57.88	66.06	66.01	65.99
Soybean Meal, Dehulled solvent	23.07	18.59	18.59	18.59
Calcium Carbonate	9.83	9.80	9.80	9.80
Dried Distillers Grains and Solubles	5.00	2.56	2.56	2.56
Animal Fat	2.28	0.73	0.73	0.73
Dicalcium Phosphorus	1.01	1.13	1.13	1.13
Salt	0.326	0.349	0.349	0.349
DL-Methionine 98.5%	0.217	0.223	0.223	0.223
L-Lysine 78.8%	0.168	0.254	0.254	0.254
DSM Trace Mineral Premix ^2^	0.120	0.120	0.120	0.120
L-Threonine 98.5%	0.048	0.091	0.091	0.091
DSM Vitamin Premix ^3^	0.038	0.038	0.038	0.038
L-Valine	0.010	0.059	0.059	0.059
Precision biotic (kg/tonne)	0.00	0.00	0.500	0.700
Calculated Values (%) [dAmino Acids:dLysine]				
Metabolizable Energy (kcal/kg)	2844	2844	2844	2844
Moisture	10.97	11.21	11.21	11.21
Crude Protein	17.50	15.50	15.50	15.50
Crude Fat	4.96	3.49	3.49	3.49
Calcium	4.20	4.20	4.20	4.20
Crude Fiber	2.15	2.02	2.02	2.02
Available Phosphorus	0.45	0.45	0.45	0.45
Choline (mg/kg)	1531.22	1378.51	1378.51	1378.51
Dig. Lys	0.90	0.85	0.85	0.85
Dig. Arg	0.99 [110.09]	0.85 [100.00]	0.85 [100.00]	0.85 [100.00]
Dig. TSAA	0.72 [80.00]	0.68 [80.00]	0.68 [80.00]	0.68 [80.00]
Dig. Val	0.72 [80.00]	0.68 [80.00]	0.68 [80.00]	0.68 [80.00]
Dig. Ile	0.64 [70.66]	0.55 [64.67]	0.55 [64.67]	0.55 [64.67]
Dig. Thr	0.60 [66.67]	0.57 [67.06]	0.57 [67.06]	0.57 [67.06]
Dig. Met	0.47 [52.62]	0.45 [53.45]	0.45 [53.45]	0.45 [53.45]
Dig. Trp	0.17 [19.04]	0.15 [17.07]	0.15 [17.07]	0.15 [17.07]
Analyzed Values (%)				
Moisture	10.55	10.55	10.49	10.83
Crude Protein	16.50	14.68	14.88	14.71
Crude Fat	5.30	3.71	3.92	3.98
Crude Fiber	2.47	2.24	2.09	2.43

^1^ treatments: PC = 17.5% crude protein (CP); NC = 15.5% CP; NC+PB1 = 15.5% CP + 0.5 kg/ton precision biotic (PB); NC+PB2 = 15.5% CP + 0.7 kg/ton PB. ^2^ manganese—75 mg/kg; zinc—65 mg/kg; iron—50 mg/kg; selenium—250 ppm; copper—7000 ppm; iodine—1000 ppm. ^3^ contains 3.97 × 10^7^ IU/kg vitamin A, 1.32 × 10^5^ IU/kg vitamin E, and 1.20 × 10^6^ FYT/kg phytase; also contains biotin, folic acid, menadione, niacin, d-pantothenic acid, riboflavin, thiamine, vitamin B_6_, vitamin B_12,_ and vitamin D_3_.

**Table 2:** Effect of varying levels of a precision biotic in a reduced crude protein diet on egg performance data.

Trt ^1^	HDEP ^2^ (%)	AEW ^3^ (g)	UE ^4^ (%)
PC	98.75 ^a^	59.17	4.81
NC	97.52 ^b^	58.26	5.14
NC+PB1	97.92 ^ab^	58.64	5.06
NC+PB2	98.19 ^ab^	58.70	4.58
SEM ^5^	0.2289	0.2528	0.5140
*p*-value	0.0028	0.0935	0.8631

^a,b^ means within a column lacking a common superscript differ (*p* ≤ 0.05) as per least significant difference. Abbreviations: ^1^ Trt = treatment; PC = 17.5% CP; NC = 15.5% CP; NC+PB1 = 15.5% CP + 0.5 kg/ton precision biotic (PB); NC+PB2 = 15.5% CP + 0.7 kg/ton PB; ^2^ HDEP = hen–day egg production; ^3^ AEW = average egg weight; ^4^ UE = unsalable egg (shell-less or soft-shelled, extremely small, extremely large, dirty, cracked, bloody, or very visible ridges or calcium deposits; ^5^ SEM = standard error of means. n= 504 observations per treatment.

**Table 3:** Effect of varying levels of a precision biotic in a reduced crude protein diet on feed conversion data.

Trt ^1^	gf/ge ^2^ (g)	kg/doz ^3^ (kg)	g/b/d ^4^ (g)	lbs/100 ^5^ (lbs)	CBW ^6^ (g)
PC	2.002	1.402	115.14	25.38	564.95
NC	2.056	1.431	115.92	25.56	377.36
NC+PB1	2.026	1.415	115.31	25.42	479.18
NC+PB2	2.020	1.419	115.79	25.53	478.29
SEM ^7^	0.0226	0.0074	0.5520	0.1217	54.71
*p*-value	0.2355	0.0624	0.7042	0.7043	0.2473

Abbreviations: ^1^ Trt = treatment; PC = 17.5% CP; NC = 15.5% CP; NC+PB1 = 15.5% CP + 0.5 kg/ton precision biotic (PB); NC+PB2 = 15.5% CP + 0.7 kg/ton PB; ^2^ gf/ge = grams of feed consumed per gram of egg produced; ^3^ kg/doz = kilograms of feed consumed per dozen eggs produced; ^4^ g/b/d = grams of feed per bird per day; ^5^ lbs/100 = pounds of feed per hundred birds per day; ^6^ CBW = change in body weight; ^7^ SEM = standard error of means; N for gf/ge, kg/doz, g/b/d, lbs/100 = 504 observations per treatment; N for CBW = 28 observations per treatment.

**Table 4:** Effect of varying levels of a precision biotic in a reduced crude protein diet on apparent digestibility of crude protein and nitrogen.

Trt ^1^		AID ^2^ of CP (%)	ATTD ^3^ of CP (%)
PC		58.05	43.15 ^ab^
NC		73.71	55.95 ^a^
NC+PB1		72.18	41.32 ^b^
NC+PB2		70.78	37.69 ^b^
SEM ^4^		7.935	5.833
*p*-value		0.2651	0.0462
Pairwise Comparisons (*p*-Values)			
PC vs.	NC NC+PB1	0.0813 0.1130	0.0549 0.7726
	NC+PB2	0.1507	0.3930
NC vs.	NC+PB1	0.8589	0.0306
	NC+PB2	0.7337	0.0090
NC+PB1 vs.	NC+PB2	0.8707	0.5678

^a,b^ means within a column lacking a common superscript differ (*p* ≤ 0.05) as per least significant difference. Bolded *p*-values are significant. Abbreviations: ^1^ Trt = treatment; PC = 17.5% CP; NC = 15.5% CP; NC+PB1 = 15.5% CP + 0.5 kg/ton precision biotic (PB); NC+PB2 = 15.5% CP + 0.7 kg/ton PB; ^2^ AID = apparent ileal digestibility; ^3^ ATTD = apparent total tract digestibility; ^4^ SEM = standard error of means; n = 7 observations per treatment.

**Table 6:** Economic analysis for varying precision biotic levels in a reduced crude protein diet.

Trt ^1^	Feed Cost (USD/100/d) ^2^	Income (USD/100/d)	Income–Feed Cost (USD/100/d)
PC	7.06 ^a^	29.38	22.33
NC	6.79 ^b^	28.81	22.02
NC+PB1	6.78 ^b^	29.14	22.36
NC+PB2	6.82 ^b^	29.40	22.58
SEM ^3^	0.0324	0.4200	0.4328
*p*-value	<0.0001	0.7381	0.8384

^a,b^ means within a column lacking a common superscript differ (*p* ≤ 0.05) as per least significant difference. Bolded *p*-values are significant. Abbreviations: ^1^ Trt = treatment; PC = 17.5% CP; NC = 15.5% CP; NC+PB1 = 15.5% CP + 0.5 kg/ton precision biotic (PB); NC+PB2 = 15.5% CP + 0.7 kg/ton PB; ^2^ USD/100/d = dollars per hundred birds per day; ^3^ SEM = pooled standard error of means.

## Data Availability

The original contributions presented in the study are included in the article; further inquiries can be directed to the corresponding author.
